# Supplementary material for: Origin of band gaps in 3d perovskite oxides
Source: Nat Commun. 2019 Apr 10;10:1658. doi: 10.1038/s41467-019-09698-6 (PMC6458163; doi:10.1038/s41467-019-09698-6)
Supplement: Supplementary file 1 — Supplementary Information [file 41467_2019_9698_MOESM1_ESM.pdf]

SUPPLEMENTARY INFORMATION

Supplementary Note 1: The perovskite structure and the possible lattice distortions

ABO_3 oxide perovskites adopt a simple cubic structure (Pm-3m symmetry) in the ideal case based on corner-sharing O_6 octahedra located around the B site cation (Supplementary Fig.1.a). Owing to the A-to-B cation size mismatch, evaluated through the 1926 Goldschmidt tolerance factor $t = \frac{r_A + r_O}{\sqrt{2}(r_B + r_O)}$ where *r* is the ionic radius of the ions of the system [1], the perovskite can undergo structural distortions. Whereas for *t*=1 the structure is perfectly cubic, *t* is usually smaller than 1 – although larger than 0.7 – and most of the perovskites exhibits oxygen octahedra rotations. It usually results in a symmetry lowering from a cubic to an orthorhombic Pbnm cell that is characterized by out-of-phase (Supplementary Fig.1.f) and in-phase (Supplementary Fig.1.b) O_6 group rotations along the x-y and z cubic axes, respectively, namely $a^-a^-c^+$ rotations pattern following Glazer's notation [2]. In addition to O_6 rotations, they can also display antipolar AFE and AP motions (Supplementary Fig.1.c and g) and/or some octahedra deformations such as a Jahn-Teller (JT) distortion, either propagating in phase (Supplementary Fig.1.d) or in anti-phase (Supplementary Fig.1.h) along the c axis, or a breathing of oxygen cage octahedron B_{oc} (Supplementary Fig.1.e).

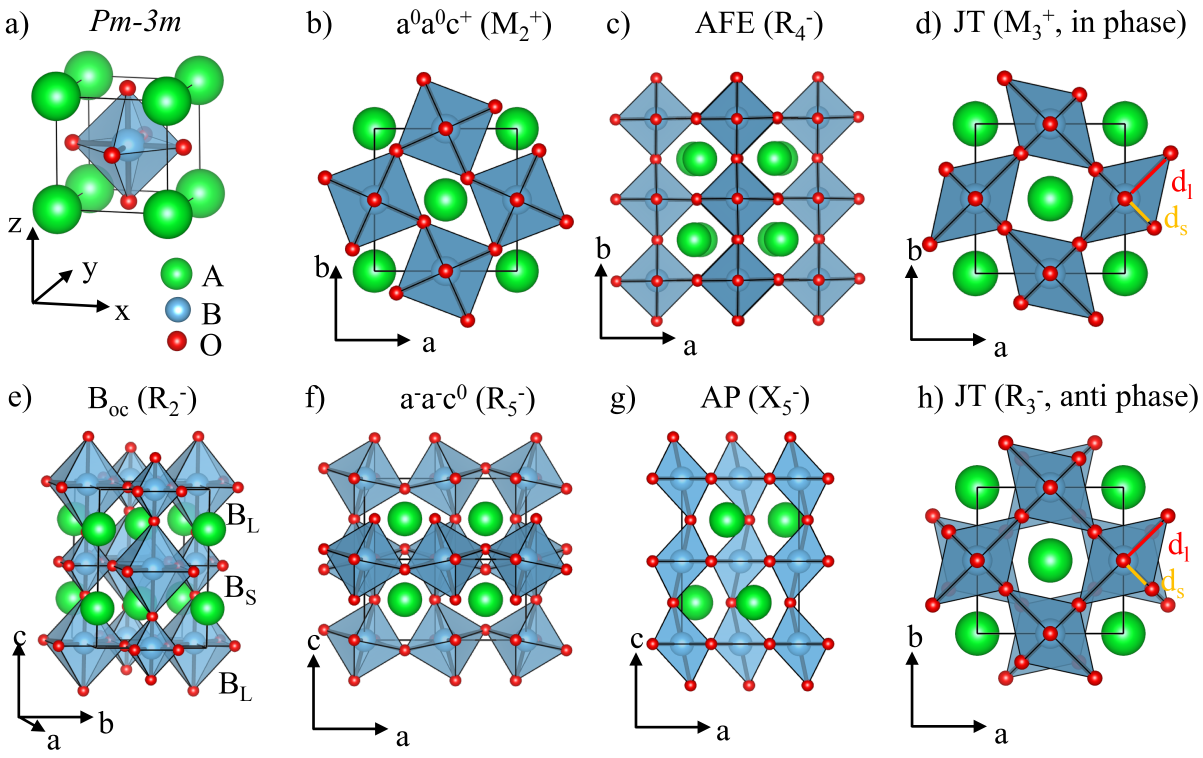

Supplementary Figure 1: Structural aspect exhibited by oxide perovskites. (a) High symmetry Pm-3m perovskite structure observed when t=1. (b-h) When $t \leq 1$, the perovskite can accommodate different lattice distortions such as oxygen cage rotations (b and f), antipolar motions AFE and AP (c and g) and octahedra deformations such Jahn-Teller mode (d and h) and/or a breathing mode B_{oc} (e). Irreducible representation of the lattice distortions is provided in parenthesis assuming A site cations located at the corner of the cubic cell.

Supplementary Note 2: The non-magnetic approximation to the paramagnetic state represents a high-energy state

A common wisdom to model paramagnetism (PM) consists to use the average value $\vec{S}_{tot}$ of the magnetic moments of the materials – *i.e* $\vec{S}_{tot} = \frac{1}{N}\sum_i \vec{s}_i$=0 for a PM phase where $\vec{s}_i$ is the local moment of the magnetic site i in the cell. Through this non-spin polarized approach – or Non-Magnetic (NM) approximation –, each transition metal element experiences the same potential $V(n(\vec{r}), m(\vec{r}) = 0)$ from surrounding atoms resulting in a so-called "monomorphous approximation", where $n(\vec{r})$ and $m(\vec{r})$ are electronic and magnetic densities respectively.

Supplementary Table 1 reports key results of the full geometry optimization (atomic positions + lattice parameters) of cubic and lower symmetry space groups using the NM approximation to PM. In the lowest energy phase within the NM approximation, all materials experimentally observed insulating in PM phases are incorrectly found metallic except $LaVO_3$ (d^2) and $CaFeO_3$ (d^4) that relax to insulating phases. For these materials, we find that electrons are spin-paired in one ($LaVO_3$) and two ($CaFeO_3$) d orbitals causing a band gap between doubly occupied and unoccupied t_{2g} states, *i.e.* band insulator characteristics (Supplementary Fig.2). Nevertheless, this is at odds with our ground state results for these two materials where d electrons are located in different orbitals due to the Hund's rule. It results in a huge energy difference (ΔE>1eV in general) between NM and spin-ordered solutions. Furthermore, the structural relaxation for $CaFeO_3$ stabilizes an orthorhombic Pbnm symmetry rather than the experimentally observed monoclinic $P2_1/n$ symmetry in the lowest temperature PM phase (Supplementary Table 1).

	$YTiO_3$	$CaVO_3$	$SrVO_3$	$LaVO_3$	$CaMnO_3$	$LaMnO_3$	$CaFeO_3$	$LaFeO_3$	$YNiO_3$
Structure	Pbnm	Pbnm	Pm-3m	$P2_1/n$	Pbnm	Pbnm	Pbnm	Pbnm	Pbnm
ΔE_{NM-GS} (meV/f.u)	452	53	58	1177	958	4316	1977	2115	133
E_g (eV)	0	0	0	0.51	0	0	0.09	0	0
ΔE_{NM-GS} (cubic, meV/f.u)	1907	207	58	1650	1139	4713	2450	3814	929
$E_{g,cubic}$ (eV)	0	0	0	0	0	0	0	0	0

Supplementary Table 1: Energy differences between the nonmagnetic solutions (fully relaxed and relaxed cubic phase) and the spin-polarized ground state identified in our DFT calculations (in meV/f.u) for the different compounds. Band gaps E_g (in eV) and identified space groups of the relaxed structures are provided.

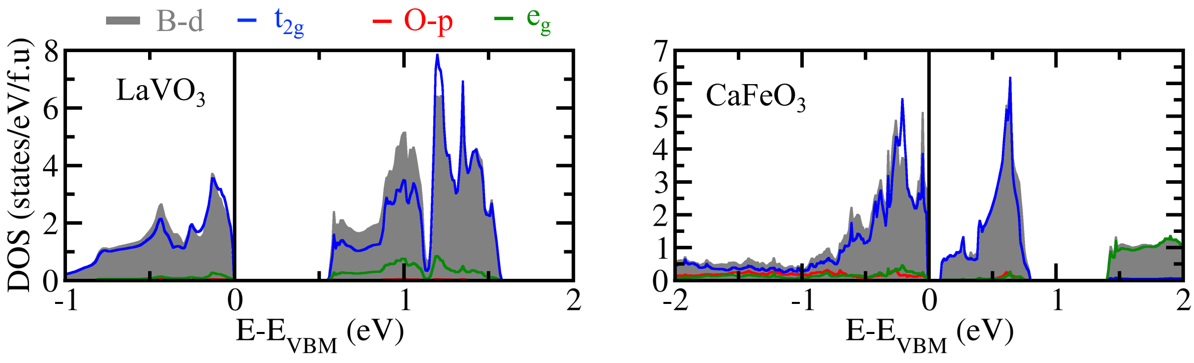

Supplementary Figure 2: Projected density of states on B d levels (grey) and O p states (red) in $LaVO_3$ (B=V, left panel) and $CaFeO_3$ (B=Fe, right panel). Splitting on t_{2g} (blue) and e_g (green) levels are provided.

<u>Supplementary Note 3: Method details</u>

DFT parameters*:* Density functional Theory calculations were performed with the Vienna Ab initio Simulation (VASP) package [3,4]. We have employed the PBEsol functional [5] plus a *U* potential on the TM d levels. We have used a single effective U_{eff} = *U-J* parameter for all compounds (Dudarev *et al* method [6]) except for $LaMnO_3$ where both *U* and *J* parameters enter separately (Lichtenstein *et al* method [7]). *U* and *J* values were copied from past literature (see Supplementary Table 2) and they were usually providing the best agreement on the low temperature phase properties or they were fitted for remaining materials. We do not claim that these values have to be taken for granted and one may probably fit better values to

increase agreement with experimental data. This is beyond the scope of the present paper. We have used Projected Augmented Wave (PAW) pseudopotentials for all ions [8]. Most notably, only 4s and 3d electrons are considered as valence electrons for 3d transition metal elements. The plane wave cut-off energy was set to 500 eV. The k-point mesh was set to 8*8*8 for the cubic cell, 6*6*4 for orthorhombic and monoclinic cells and 3*3*2 for the PM supercells. Full geometry relaxations were performed until forces were lower than 0.005 eV. $Å^{-1}$.

	$YTiO_3$	$CaVO_3$	$SrVO_3$	$LaVO_3$	$CaMnO_3$	$LaMnO_3$	$CaFeO_3$	$LaFeO_3$	$YNiO_3$
U/J parameters (eV)	2.5/0 [9]	1.25/0	1.25/0	3.5/0 [10]	0/0 [11]	8/1.9 [12]	3.8/0 [13,14]	3.8/0 [13]	2/0 [15]

Supplementary Table 2*:* *U* and *J* parameters (in eV) used in the simulations.

Explored magnetic states and crystal structures: Structural relaxations were performed with FM, AFM (A, C, G and S) and PM orders with spins only considered at the collinear level. We started from the high symmetry cubic phase (Pm-3m), as well as orthorhombic (Pbnm) and monoclinic ($P2_1/b$ and $P2_1/n$) symmetries. Symmetry of our AFM/FM (PM) optimized structures was extracted using the FINDSYM software [16] with tolerances of 0.0005 (0.08) on atomic positions and lattice parameters for spin-ordered (PM) phases.

d orbital occupation matrix: In order to accelerate the PM calculation, we initially imposed the d orbital occupancies to the solver and converge the calculations using the modified VASP routine discussed in reference [17]. For insulating materials, the d orbital occupancies were unfrozen and the DFT self-consistent field as well as geometrical relaxation were left to perform. For metals, the orbital occupancies were left frozen in order to avoid spin flips and spontaneous decay to disproportionating phase in $CaFeO_3$ and $RNiO_3$ compounds. Since rotations and octahedra deformations strongly mix "cubic-d" orbitals together, we have extracted the d orbital occupancies matrix elements by running a preliminary ferromagnetic calculation and flipped manually the desired spins. For runs with orbital broken symmetry (OBS) in cubic phases, we directly forced specific d orbital occupancies by one or zero electron and we pre-converged a solution. The solver was then allowed to update occupation matrices.

Supplementary Note 4: Special Quasirandom Structures (SQS) details

The SQS was modelled using the ATAT package [18,19] and we imposed to transition metal sites to possess either a spin up or a spin down (spins were only considered at the colinear level). The disorder was generated independently for each Wyckoff position for disproportionating materials.

In order to have a sufficiently large number of TM sites in our PM phase, we have considered 160 atoms supercells (32 transition metal sites) corresponding to the orthorhombic (monoclinic) cell where all directions are multiplied by 2 -- $(2\sqrt{2}a, 2\sqrt{2}a, 4a)$ cubic cell. We have checked the convergence of energies, magnetic moments and band gaps as a function of the number of magnetic sites in $YTiO_3$ and $YNiO_3$ (Supplementary Table 3).

	$YTiO_3$			$YNiO_3$		
N	m_{Ti} (μ_B)	ΔE (meV/f.u)	E_g (eV)	m_{Ni} (μ_B)	ΔE (meV/f.u)	E_g (eV)
40	0.897	0.00	1.17	1.311/0.148	0.00	0.281
80	0.896	-13	1.21	1.279/0.089	-6.77	0.320
160	0.896	-33	1.27	1.262/0.162	-9.44	0.496
320	-	-	-	1.265/0.001	-9.31	0.359

Supplementary Table 3*:* Convergence of magnetic moments m (in μ_B), energies difference *wrt* 40 atom unit cell (in meV/f.u) and band gap E_g (in eV) as a function of the number N of transition metal sites considered in the PM-SQS cell for $YTiO_3$ and $YNiO_3$.

We report in tables 4 and 5 the atomic positions of the B site cation bearing a spin up and a spin down for compounds without (Supplementary Table 4) and with (Supplementary Table 5) disproportionation effects.

B - spin up	0.25	0.75	0.00	B - spin down	0.25	0.75	0.50
	0.00	0.50	0.00		0.00	0.50	0.50
	0.25	0.25	0.00		0.00	0.00	0.50
	0.00	0.00	0.00		0.50	0.50	0.00
	0.25	0.25	0.50		0.75	0.75	0.50
	0.75	0.75	0.00		0.50	0.00	0.00
	0.50	0.50	0.50		0.75	0.25	0.50
	0.75	0.25	0.00		0.50	0.00	0.75
	0.50	0.00	0.50		0.25	0.75	0.75
	0.50	0.50	0.75		0.50	0.00	0.25
	0.50	0.50	0.25		0.25	0.75	0.25
	0.25	0.25	0.25		0.25	0.25	0.75
	0.00	0.00	0.75		0.75	0.75	0.75
	0.75	0.25	0.75		0.00	0.00	0.25
	0.00	0.50	0.25		0.75	0.75	0.25
	0.75	0.25	0.25		0.00	0.50	0.75

Supplementary Table 4: *Atomic positions of B cations in the SQS cubic phase of non disproportionating compounds. The B cation is located in (0,0,0) in the parent 5 atom cubic cell.*

B1 - spin up	0.75	0.75	0.75	B2 - spin up	0.75	0.75	0.50
	0.25	0.75	0.25		0.75	0.75	0.00
	0.75	0.25	0.75		0.25	0.75	0.00
	0.25	0.25	0.25		0.75	0.25	0.50
	0.50	0.00	0.00		0.75	0.25	0.00
	0.00	0.00	0.50		0.25	0.25	0.50
	0.50	0.50	0.00		0.50	0.50	0.25
	0.00	0.50	0.50		0.00	0.50	0.75
B1 - spin down	0.75	0.75	0.25	B2 - spin down	0.25	0.75	0.50
	0.25	0.75	0.75		0.25	0.25	0.00
	0.75	0.25	0.25		0.50	0.00	0.25
	0.25	0.25	0.75		0.50	0.00	0.75
	0.50	0.00	0.50		0.00	0.00	0.25
	0.00	0.00	0.00		0.00	0.00	0.75
	0.50	0.50	0.50		0.50	0.50	0.75
	0.00	0.50	0.00		0.00	0.50	0.25

Supplementary Table 5*:* Atomic positions of B cations in the SQS cubic phase of disproportionating compounds. The A cation is located in (0,0,0) in the parent 5 atom cubic cell.

Supplementary Note 5: Energies of the different magnetic phases

We summarize in Supplementary table 6 the energy difference of the different magnetic phases (relaxed structures) *wrt* the ferromagnetic solution (in meV/ f.u) for the different compounds. For $LaVO_3$, both PM and AFMG orders relax to an orthorhombic Pbnm symmetry even when starting from a $P2_1/b$ space group.

	Space group	FM	AFMA	AFMC	AFMG	AFMS	PM
$CaVO_3$	*Pbnm*	0.00	-217.06	-215.39	53.20	-	-253.22
$SrVO_3$	*Pbnm*	0.00	120.08	-2.98	-1.93	-	-62.62
$YTiO_3$	*Pbnm*	0.00	3.53	16.61	18.67	-	9.62
$LaVO_3$	*$P2_1/b$*	0.00	-10.92	-15.69	-13.94 (*Pbnm*)	-	-6.30 (*Pbnm*)
	Pbnm	11.06	3.60	-12.83	-13.94	-	-6.30
$CaMnO_3$	*Pbnm*	0.00	-46.17	-63.23	-72.07	-	-49.44
$LaMnO_3$	*Pbnm*	0.00	-0.38	9.35	3.49	-	3.10
$CaFeO_3$	*$P2_1/n$*	0.00	172.48	102.59	49.49	34.52	51.36
	Pbnm	1.09	57.71	115.22	152.60	-	68.78
$LaFeO_3$	*Pbnm*	0.00	-93.01	-183.52	-257.22	-	-139.36
$YNiO_3$	*$P2_1/n$*	0.00	11.56	11.50	58.09	-1.27	2.51
	Pbnm	3.44	27.89	54.78	56.49	-	24.77

Supplementary Table 6: Energy difference of the different magnetic phases (relaxed structures) *wrt* the ferromagnetic solution (in meV/f.u) for the different compounds. When two space groups are reported for a compound, the energy difference is provided wrt the lowest energy phase with the FM order. The relaxed space group is provided in parenthesis when it is different than the initially imposed one.

Supplementary Note 6: Symmetry mode analysis

In order to extract the amplitude (in Å) of pure lattice distortions appearing in our relaxed ground states, we performed symmetry mode analysis of our structures with the PM and (A-)FM orders using the Amplimodes application [20,21] available on the Bilbao

Crystallographic Server. The reference structure was set to a high symmetry Pm-3m cubic structure whose lattice parameter was optimized using a FM order for each material. Amplitudes of lattice distortions appearing in the perovskite are reported in Supplementary Table 7. We also report experimental values for comparison, using crystallographic structures reported in literature, either in the PM and/or the spin-ordered phase.

Our relaxed materials reproduce the amplitude of experimentally observed distortions, showing that DFT+*U* is able to capture the structural properties of oxide perovskites. Most notably, distortions intimately connected to electronic and magnetic degrees of freedom (Jahn-Teller, breathing mode B_{oc}) are properly reproduced in $LaVO_3$, $CaFeO_3$ and $YNiO_3$. Finally, we observe that the NM approximation ultimately misses to capture the bond disproportionation effect in $CaFeO_3$ and $YNiO_3$. We emphasize that $SrVO_3$ is not presented since it does not develop any lattice distortions, *i.e.* it remains cubic.

		$a^-a^-c^0$ (R_5^-)	$a^0a^0c^+$ (M_2^+)	AP (X_5^-)	AFE (R_4^-)	JT (M_3^+)	JT (R_3^-)	B_{oc} (R_2^-)
$CaMnO_3$	Exp (PM 302 K [22])	0.98	0.80	0.36	0.11	0.04	-	-
	AFMG (DFT+U)	1.06	0.82	0.44	0.08	0.01	-	-
	PM (DFT+U)	1.08	0.84	0.45	0.09	0.01	-	-
	NM (DFT+U)	1.02	0.81	0.43	0.09	0.01	-	-
$LaFeO_3$	Exp (AFM-G 300 K [23])	1.34	0.67	0.39	0.24	0.00	-	-
	AFMG (DFT+U)	1.24	0.76	0.42	0.09	0.01	-	-
	PM (DFT+U)	1.26	0.79	0.43	0.09	0.01	-	-
	NM (DFT+U)	0.99	0.59	0.29	0.06	0.01	-	-
$YTiO_3$	Exp (PM 290 K [24])	1.82	1.30	0.92	0.24	0.00	-	-
	FM (DFT+U)	1.97	1.35	1.00	0.29	0.02	-	-
	PM (DFT+U)	1.97	1.35	1.00	0.30	0.02	-	-
	NM (DFT+U)	1.92	1.29	1.00	0.26	0.09	-	-
$LaMnO_3$	Exp (PM 300 K [25])	1.18	0.84	0.16	0.08	0.30	-	-
	AFMA (DFT+U)	1.31	1.01	0.67	0.12	0.37	-	-
	PM (DFT+U)	1.31	1.03	0.68	0.12	0.38	-	-
	NM (DFT+U)	1.19	0.54	0.27	0.14	0.00	-	-
$CaVO_3$	Exp (PM 300 K [26])	0.88	0.75	0.31	0.03	0.01	-	-
	PM (DFT+U)	1.01	0.79	0.43	0.08	0.01	-	-
$LaVO_3$	Exp (AFM-C 10 K $P2_1/b$ [27])	1.16	0.75	0.39	0.05	0.01	0.08	0.00
	Exp (PM 298 K Pbnm [28])	1.16	0.67	0.37	0.12	0.08	-	-
	AFMC (DFT+U)	1.30	0.88	0.52	0.12	0.03	0.09	0.00
	PM (DFT+U)	1.31	0.90	0.54	0.13	0.07	-	-
	NM (DFT+U)	1.25	1.01	0.70	0.10	0.12	0.12	0.02
$CaFeO_3$ ($P2_1/n$)	Exp (PM 15 K $P2_1/n$ [29])	1.06	0.82	0.40	0.11	0.04	0.00	0.18
	FM (DFT+U)	1.08	0.79	0.46	0.09	0.00	0.00	0.07
	PM (DFT+U)	1.09	0.81	0.47	0.09	0.01	0.00	0.14
$CaFeO_3$ (Pbnm)	Exp (PM 300 K Pbnm [29])	0.99	0.78	0.36	0.05	0.01	-	-
	NM (DFT+U)	1.16	0.91	0.49	0.10	0.09	-	-
	PM (DFT+U)	1.07	0.80	0.46	0.09	0.02	-	-
$YNiO_3$ ($P2_1/n$)	Exp (PM 300 K $P2_1/n$ [30])	1.47	1.14	0.82	0.19	0.05	0.00	0.13
	AFMS (DFT+U)	1.45	1.11	0.82	0.21	0.05	0.00	0.14
	PM (DFT+U)	1.45	1.11	0.82	0.21	0.05	0.00	0.14
$YNiO_3$ (*Pbnm*)	PM (DFT+U)	1.43	1.12	0.83	0.22	0.02	-	-
	NM (DFT+U)	1.40	1.08	0.81	0.20	0.04	-	-

Supplementary Table 7*:* Symmetry adapted modes providing the amplitude of distortions (in Å) described in Supplementary Fig.1 for the different compounds in their LT and HT-PM phases. Experimental data available in literature are provided for comparison.

Supplementary Note 7: Orbital patterns induced by in-phase and anti-phase Jahn-Teller modes

Two types of Jahn-Teller motions are observed in our tested oxide perovskites. The octahedra are always distorted with two B-O bonds contractions while the two others are elongated, with opposite motions on all neighboring site in the (xy)-plane (Supplementary Fig.1.d). Nevertheless, the motion can be identical between consecutive planes along the z direction (Supplementary Fig.1.d) or in anti-phase (Supplementary Fig.1.h), therefore producing distinct symmetries. Consequently, two different orbital patterns can be observed depending on the JT motion. In the two cases, the e_g electron/2nd t_{2g} electron in $LaMnO_3$/$LaVO_3$ occupies an orbital with a shape that is pointing to different directions on neighboring B sites in the (xy)-plane (Supplementary Fig.3.a and c). When going to consecutive planes along the z direction, the shape of the orbital is either identical for an in-phase JT motion ($LaMnO_3$, Supplementary Fig.3.b) or alternating for an anti-phase JT motion ($LaVO_3$, Supplementary Fig.3.d). For the latter case, it yields a rock-salt like pattern of occupied orbitals.

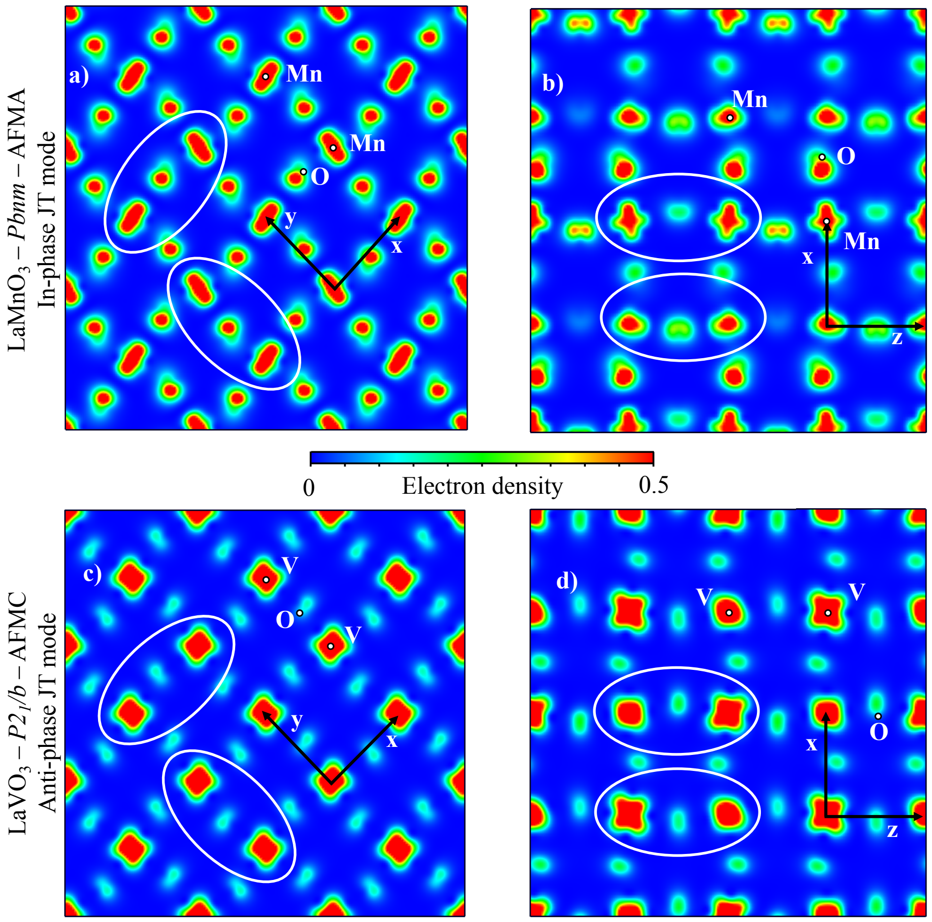

Supplementary Figure *3*: Partial charge density maps of the latest two occupied bands in $LaMnO_3$ (a,b) and $LaVO_3$ (c,d) ground state, illustrating the role of in-phase and anti-phase Jahn-Teller motions.

Supplementary Note 8: Gapping mechanism in $CaFeO_3$

We have checked that the mechanism opening the band gap in $YNiO_3$ also applies to $CaFeO_3$. We start from a cubic phase and we artificially break Fe e_g level degeneracies by nudging electrons in a rock-salt pattern with either half-filled or empty e_g states on neighboring Fe sites (*e.g.* (1,1) and (0,0) on degenerate e_g partners). After the variational self-consistency, the compound reaches a lower energy state (ΔE=-43 meV/f.u *wrt* the cubic phase with degenerate levels) by producing two distinct electronic structures on neighboring Fe sites, one site (Fe_L) bearing a larger magnetic moment than the other site (Fe_S) (Supplementary Fig.4.d). On the contrary to $YNiO_3$, we observe that the orbital broken symmetry (OBS) is able by itself to open a gap in the cubic phase (Supplementary Fig.4.a). The electronic structure is unstable in $CaFeO_3$ and is likely distorting to produce two local environments for Fe cations, one with half-filled and one with empty e_g states.

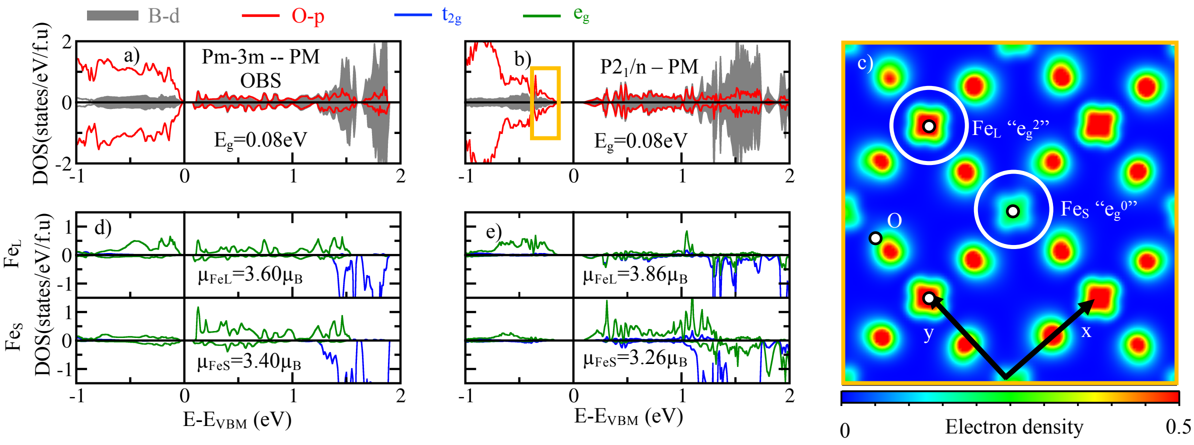

Supplementary Figure 4*:* (a,b) Averaged projected density of states on Fe d levels (grey) and O p levels (red) in $CaFeO_3$ in the cubic (a) and monoclinic (b) PM phase. (d,e) Projected density of states on t_{2g} (blue) and e_g (green) levels for a couple of Fe_L (upper panel) and Fe_S (lower panel) cations within the supercells. c) Partial charge density maps in the (ab)-plane of levels located near the Fermi level reported in b.

Relaxing the structure starting form this cubic phase with orbital broken symmetries produces a "$P2_1/n$" symmetry. We observe the appearance of the breathing mode, that produces the two electronic structures for Fe cations, characterized by two very distinct magnetic moments (Supplementary Fig.4.e). This suggests high-spin Fe_L^{3+} and Fe_S^{5+} cations, a fact confirmed by the partial charge density map reported in Fig.5.c in which we see that electrons are more localized on Fe_L sites than on Fe_S cations. Finally, the compound is insulating with a band gap of roughly 0.08 eV, similar to that exhibited in the cubic phase with OBS (Supplementary Fig.4.b).

Therefore, $CaFeO_3$ undergoes disproportionation effect of the unstable 4+ formal oxidation state (FOS) of Fe ions that transforms to 3+/5+ more stable FOS. We again note that owing to a charge self-regulation mechanism, physical charges around both Fe cations are nearly constant. It is worth to emphasize that although disproportionation effect would produce a gap in the cubic phase, steric effects first strongly stabilizes an orthorhombic symmetry (ΔE=-1894 mev/f.u *wrt* to cubic phase with (1/2,1/2) e_g occupancies) that has no disproportionation effect and *de facto* no gap.

Supplementary Note 9: Additional details on disproportionating ABO_3 compounds

We have performed additional simulations for $GdNiO_3$, $SmNiO_3$, $NdNiO_3$ and $PrNiO_3$ using the PM and AFM orders both in the orthorhombic and monoclinic phases. We report in Table 8 the key quantities (energy differences, magnetic moments, band gaps, and space groups) of the relaxed geometries.

		m_{NiL} - m_{NiS} (μB)	E_g (eV)	ΔE (meV)
$GdNiO_3$	Pbnm PM	0.75	0.00	0.00
	$P2_1/n$ PM	1.25 - 0.17	0.44	-7.86
	$P2_1/n$ AFM-S	1.24 - 0	0.57	-13.16
$SmNiO_3$	Pbnm PM	0.72	0.00	0.00
	$P2_1/n$ PM	1.23 - 0.17	0.35	-12.47
	$P2_1/n$ AFM-S	1.19 - 0	0.54	-20.64
$NdNiO_3$	Pbnm PM	0.72	0.00	0.00
	$P2_1/n$ PM	1.20 - 0.18	0.21	0.62
	$P2_1/n$ AFM-S	1.177 - 0	0.49	-12.38
$PrNiO_3$	Pbnm PM	0.72	0.00	0.00
	$P2_1/n$ PM	1.18 - 0. 20	0.13	8.53
	$P2_1/n$ AFM-S	1.174 - 0	0.44	-7.46

Supplementary Table 8: Magnetic moments m (μ_B), band gap (eV) and energy difference (in meV/f.u) wrt the orthorhombic paramagnetic solution for additional $RNiO_3$ members with their AFM and PM magnetic orders.

Supplementary References

1. Goldschmidt, V. M. Die gesetze der krystallochemie. *Naturwissenschaften* **14,** 477 (1926).
2. Glazer, A. M. The classification of tilted octahedra in perovskites. *Acta Crystallogr. Sect. B Struct. Crystallogr. Cryst. Chem.* **28,** 3384 (1972).
3. Kresse, G. & Hafner, J. Ab initio molecular dynamics for liquid metals. *Phys. Rev. B* **47,** 558 (1993).
4. Kresse, G. & Furthmüller, J. Efficiency of ab-initio total energy calculations for metals and semiconductors using a plane-wave basis set. *Comput. Mater. Sci.* **6,** 15 (1996).
5. Perdew, J. P. *et al.* Restoring the Density-Gradient Expansion for Exchange in Solids and Surfaces. *Phys. Rev. Lett.* **100,** 136406 (2008).
6. Dudarev, S. L., Savrasov, S. Y., Humphreys, C. J. & Sutton, a. P. Electron-energy-loss spectra and the structural stability of nickel oxide: An LSDA+U study. *Phys. Rev. B* **57,** 1505 (1998).
7. Liechtenstein, A. I., Anisimov, V. I. & Zaanen, J. Density-functional theoy and strong

interactions: Orbital ordering in Mott-Hubbard insulators. *Phys. Rev. B* **52,** R5467 (1995).

8. Blöchl, P. E. Projector augmented-wave method. *Phys. Rev. B* **50,** 17953 (1994).
9. Varignon, J., Grisolia, M. N., Preziosi, D., Ghosez, P. & Bibes, M. Origin of the orbital and spin ordering in rare-earth titanates. *Phys. Rev. B* **96,** 235106 (2017).
10. Varignon, J., Bristowe, N. C., Bousquet, E. & Ghosez, P. Coupling and electrical control of structural, orbital and magnetic orders in perovskites. *Sci. Rep.* **5,** 15364 (2015).
11. Bhattacharjee, S., Bousquet, E. & Ghosez, P. Engineering multiferroism in $CaMnO_3$. *Phys. Rev. Lett.* **102,** 117602 (2009).
12. Mellan, T. A. *et al.* Importance of anisotropic Coulomb interaction in $LaMnO_3$. *Phys. Rev. B* **92,** 085151 (2015).
13. Zanolli, Z., Wojdeł, J. C., Íñiguez, J. & Ghosez, P. Electric control of the magnetization in $BiFeO_3$/$LaFeO_3$ superlattices. *Phys. Rev. B* **88,** 060102(R) (2013).
14. Cammarata, A. & Rondinelli, J. M. Spin-assisted covalent bond mechanism in 'charge-ordering' perovskite oxides. *Phys. Rev. B* **86,** 195144 (2012).
15. Varignon, J., Grisolia, M. N., Íñiguez, J., Barthélémy, A. & Bibes, M. Complete phase diagram of rare-earth nickelates from first-principles. *npj Quantum Mater.* **2,** 21 (2017).
16. Stokes, H. T. & Hatch, D. M. FINDSYM: Program for identifying the space-group symmetry of a crystal. *J. Appl. Crystallogr.* **38,** 237 (2005).
17. Allen, J. P. & Watson, G. W. Occupation matrix control of d- and f-electron localisations using DFT + U. *Phys. Chem. Chem. Phys.* **16,** 21016 (2014).
18. Van de Walle, A., Asta, M. & Ceder, G. The alloy theoretic automated toolkit: A user guide. *Calphad Comput. Coupling Phase Diagrams Thermochem.* **26,** 539 (2002).
19. van de Walle, A. Multicomponent multisublattice alloys, nonconfigurational entropy and other additions to the Alloy Theoretic Automated Toolkit. *Calphad Comput. Coupling Phase Diagrams Thermochem.* **33,** 266 (2009).
20. Perez-Mato, J. M., Orobengoa, D. & Aroyo, M. I. Mode crystallography of distorted structures. *Acta Crystallogr. Sect. A Found. Crystallogr.* **66,** 558–590 (2010).
21. Orobengoa, D., Capillas, C., Aroyo, I. & Perez, J. M. AMPLIMODES : Symmetry mode analysis on the Bilbao Crystallographic Server research papers. *J. Appl. Crystallogr.* **42,** 820 (2009).

22. Paszkowicz, W. *et al.* Lattice parameters and orthorhombic distortion of $CaMnO_3$. *Powder Diffr.* **25,** 46 (2010).
23. Selbach, S. M., Tolchard, J. R., Fossdal, A. & Grande, T. Non-linear thermal evolution of the crystal structure and phase transitions of $LaFeO_3$ investigated by high temperature X-ray diffraction. *J. Solid State Chem.* **196,** 249 (2012).
24. Komarek, A. C. *et al.* Magnetoelastic coupling in $RTiO_3$ (R= La, Nd, Sm, Gd, Y) investigated with diffraction techniques and thermal expansion measurements. *Phys. Rev. B* **75,** 224402 (2007).
25. Norby, P., Krogh Andersen, I. G., Krogh Andersen, E. & Andersen, N. H. The crystal structure of lanthanum manganate(iii), $LaMnO_3$, at room temperature and at 1273 K under N_2. *J. Solid State Chem.* **119,** 191 (1995).
26. Falcón, H., Alonso, J. A., Casais, M. T., Martínez-Lope, M. J. & Sánchez-Benítez, J. Neutron diffraction study, magnetism and magnetotransport of stoichiometric $CaVO_3$ perovskite with positive magnetoresistance. *J. Solid State Chem.* **177,** 3099 (2004).
27. Bordet, P. *et al.* Structural aspects of the crystallographic-magnetic transition in $LaVO_3$ around 140 K. *J. Solid State Chem.* **106,** 253 (1993).
28. Seim, H. & Fjellvag, H. Non-Stoichiometric $LaVO_3$. I. Synthesis and Physical Properties. *Acta Chem. Scand.* **52,** 1096 (1998).
29. Woodward, P., Cox, D., Moshopoulou, E., Sleight, A. & Morimoto, S. Structural studies of charge disproportionation and magnetic order in $CaFeO_3$. *Phys. Rev. B* **62,** 844 (2000).
30. Alonso, J. A., Martínez-Lope, M. J., Casais, M. T., Aranda, M. A. G. & Fernández-Díaz, M. T. Metal−Insulator Transitions, Structural and Microstructural Evolution of $RNiO_3$ (R = Sm, Eu, Gd, Dy, Ho, Y) Perovskites: Evidence for Room-Temperature Charge Disproportionation in Monoclinic $HoNiO_3$ and $YNiO_3$. *J. Am. Chem. Soc.* **121,** 4754 (1999).
